# Validating the Enright Forgiveness Inventory in Ecuador

**DOI:** 10.1002/pchj.740

**Published:** 2024-02-15

**Authors:** Francisca Fariña, Xiang Zhao, Mercedes Novo, Gabriela Acurio

**Affiliations:** ^1^ UNESCO Chair in Transformative Education: Science, Communication and Society, University of Vigo Pontevedra Spain; ^2^ School of Behavioural, Social and Legal Sciences, Örebro University Örebro Sweden; ^3^ Forensic Psychology Institute, Faculty of Psychology, University of Santiago de Compostela Santiago de Compostela Spain

**Keywords:** Ecuador, Enright Forgiveness Inventory–30 (EFI‐30), forgiveness, psychometrics, validation

## Abstract

Research on forgiveness is limited in Ecuador. This study validated the Enright Forgiveness Inventory–30 (EFI‐30) among 960 participants in Ecuador, resulting in robust reliability and validity values. Our findings provide avenues for future research and practices.

Forgiveness is a complex of psychological changes involving attitudes, emotions, and behaviors, although varied definitions exist (for a review, see Freedman, 2022). Forgiveness constitutes a psychological strength that can facilitate personal well‐being, positive emotions, and social support. Furthermore, the benefits of forgiveness work for both the offended person and the offender (Fariña & Oyhamburu, 2021).

The Enright Forgiveness Inventory (EFI; Subkoviak et al., 1995), a scale developed by Robert Enright and the Human Development Studies Group, is one of the most widely used interpersonal forgiveness measures globally. The EFI measures one's attitudes toward forgiveness; therefore, it assumes three components of attitudes, namely, affective, cognitive, and behavioral. Accordingly, six factors can be obtained: positive affect, positive cognition, positive behavior, negative affect, negative cognition, and negative behavior. The original EFI included 60 items and was later reduced to 30 items (EFI‐30). Recently, Enright et al. (2022) validated the EFI‐30 in eight regions, finding a generally high reliability across cultures. This validation work, however, did not include Latin‐American countries, such as Ecuador.

The present study aimed to validate the EFI‐30 in Ecuador. Forgiveness as a specific research topic is limited in Ecuador. Given the hegemonic presence of Christianity in the society, we could speculate on the salience and divinity of forgiveness in the Ecuadorian community. This partly explains, for example, that some familial sexual abuse among its members can be forgiven (Montenegro‐Pasquel & Pinos‐Montenegro, 2021). As a starting point, it was necessary to establish a useful instrument for measuring forgiveness for both clinical practice and academic research. Four academics from Ecuador evaluated the linguistic appropriateness of the Spanish version of the EFI‐30 conducted by Fariña et al. (2023; this version has already been validated in Spain) and deemed no further modification was needed. Thus, the present study aimed to validate the EFI‐30 among the Ecuadorian population.

For data collection, we used a snowball sampling approach. Specifically, students studying for a master's degree in legal psychology at the Pontificia Universidad Católica del Ecuador were invited. Informed of the objective of the research and the voluntary and anonymous nature of participation, the participants were also encouraged to share the survey with others in their networks. All participants in this report gave their consent to participate and provide information for scientific purposes.

We recruited 1039 participants. However, using the pseudo‐forgiveness items as a screening criterion (i.e., removing cases with a sum score ≥ 20), a final sample of 960 participants resulted. All participants are originally from Ecuador. The mean age of the sample was 34.0 (*SD* = 10.7) years. Most participants were women (60.7%), had received/were receiving a tertiary education (84.8%), and were single (65.1%). For details, see Supplementary Materials (Table S1).

The measures included three sections: EFI‐30, social desirability (Gutiérrez et al., 2016), and demographic information. For full descriptions, see Supplementary Materials, Table S2.

We used several psychometric methods to investigate the reliability and validity of the EFI‐30. Specifically, Cronbach's alpha (α), Guttman's lambda 6 (G6), and item–total correlations were used to access internal consistency; also, content validity was tested using confirmatory factor analysis (CFA) regarding forgiveness as a six‐dimensional construct (cutoff criteria for fit indices followed the recommendation by Hu & Bentler, 1999). Two correlational tests were further performed to establish criterion validity: First, associations between the EFI items and the one‐item forgiveness score were calculated; second, associations between the EFI items and the social desirability total score were calculated as per a previous study (Enright et al., 2022). Data analysis was conducted in R. CFA estimation was conducted with the lavaan package, with FIML missing data treatment and the MLR estimator.

As shown in Table S3, all items were strongly associated with their subscale total score (*r*s ranged between .69 and .92). Both Cronbach's *α* (>.85) and G6 (>.85) showed excellent values for each subscale, as summarized in Table 1.

**TABLE 1:** Standardized Factor Loadings and Internal Consistency Indices of the Enright Forgiveness Inventory–30.

Subscale	Item number and name	Λ	*r*
Positive Affect (*α* = .92; G6 = .91)	1. Warm	.66	.79
	2. Tender	.77	.86
	7. Caring	.93	.92
	8. Affection	.94	.91
	9. Friendly	.85	.88
Negative Affect (*α* = .86; G6 = .86)	3. Unloving	.63	.73
	4. Repulsed	.88	.89
	5. Cold	.85	.87
	6. Dislike	.87	.88
	10. Disgust	.61	.69
Positive Behavior (*α* = .91; G6 = .90)	11. Show friendship	.76	.83
	16. Lend him/her a hand	.78	.84
	17. Establish good relations with him/her	.82	.86
	19. Do a favor	.90	.90
	20. Aid him/her when in trouble	.91	.91
Negative Behavior (*α* = .92; G6 = .92)	12. Avoid	.83	.88
	13. Ignore	.86	.90
	14. Neglect	.90	.90
	15. Not attend to him/her	.86	.87
	18. Stay away	.79	.85
Positive Cognition (*α* = .91; G6 = .90)	22. Of good quality	.84	.86
	25. A good person	.86	.88
	27. Wish him/her well	.77	.86
	29. Think favorably of him/her	.85	.86
	30. Hope he/she succeeds	.80	.87
Negative Cognition (*α* = .89; G6 = .88)	21. Horrible	.86	.87
	23. Dreadful	.89	.89
	24. Worthless	.88	.89
	26. A bad person	.77	.85
	28. Disapprove of him/her	.59	.72

*Note*: *N* = 957. Λ = standardized factor loading; *r* = corrected item–total subscale correlation; *α* = Cronbach's *α*; and G6 = Guttman's lambda 6. Three cases with complete missing data were removed from this analysis. All factor loadings and item–total correlations showed a statistical significance (i.e., *p* < .01).

The overall six‐dimensional model showed an adequate fit during CFA: robust root‐mean‐square error of approximation = .08, 90% confidence interval [.073, .081], robust comparative fit index/ Tucker–Lewis index= .91/.90, standardized root‐mean residual = .07. The standardized factor loadings ranged from .59 to .94 (Table 1).

As shown in Table S3, all subscale total scores showed a significant association with one‐item forgiveness, indicating an established criterion validity. Judging from the associations with social desirability, only positive affect and positive cognition showed positive and significant correlations.

Our findings importantly validate the EFI‐30 in Ecuador. Based on robust internal consistency and validity values, the EFI‐30 could serve as a useful instrument in evaluating people's forgiveness in Ecuador. Our findings cohere with Enright et al.'s (2022) results from multiple cultures. Given the emerging application of forgiveness in therapy and relational counselling (Fariña & Oyhamburu, 2021), the EFI‐30 could be used alongside therapy sessions to monitor a client's changes in forgiveness. It could also be used in therapeutic jurisprudence to examine the relationship between psychological well‐being and forgiveness (Fariña et al., 2023).

The student‐centered sampling is the major limitation of our study. Our findings do not well represent, for example, residents in Ecuador with lower education levels. Future studies may consider using stratified sampling methods. Since Ecuador is a multicultural and multiethnic country, future studies could also investigate the differences in subgroups. In line with previous multinational studies (Enright et al., 2022), positive affect and positive cognition were associated, albeit weakly, with social desirability, indicating a recommendation for further cultural sensitivity investigations. Other measures, such as depression, anxiety, and anger, could also be included to establish the criterion validity of the scale. Nonetheless, our study fills a knowledge gap and provides a useful instrument for future research in forgiveness studies.

## CONFLICT OF INTEREST STATEMENT

The authors declare there are no conflicts of interest.

## ETHICS STATEMENT

Before data collection, the study was approved by the Research Ethics Commission of the Faculty of Education and Sports Sciences, University of Vigo, Spain (11–250322).

## Supporting information


**TABLE S1:** Demographic Information of Participants (*N* = 960).
**TABLE S2:** Measures in Detail.
**TABLE S3:** Correlations Between Subscales and the One‐Item Forgiveness and Social Desirability Measures.
