# Supplementary material for: Validating the Enright Forgiveness Inventory in Ecuador
Source: Psych J. 2024 Feb 15;13(4):692–4. doi: 10.1002/pchj.740 (PMC11317186; doi:10.1002/pchj.740)
Supplement: Supplementary file 1 — TABLE S1: Demographic Information of Participants (N = 960). TABLE S2: Measures in Detail. TABLE S3: Correlations Between Subscales and the One‐Item Forgiveness and Social Desirability Measures. [file PCHJ-13-692-s001.docx]

**Supplementary Materials**

**Table S1**

*Demographic Information of Participants (*N *= 960)*

| Variable | Frequency (%) |
| --- | --- |
| Age – *M*/*SD*/range, year | 34.0/10.7/18–90 |
| Sex |  |
| Man | 373 (38.9) |
| Woman | 583 (60.7) |
| Prefer not to say | 4 (0.4) |
| Gender |  |
| Male | 369 (38.6) |
| Female | 578 (60.4) |
| Other | 4 (0.4) |
| Prefer not to say | 6 (0.6) |
| Educational level |  |
| No education | 3 (0.3) |
| Primary | 6 (0.6) |
| Secondary | 137 (14.3) |
| Tertiary | 813 (84.8) |
| Relationship status |  |
| Married/cohabitating | 339 (35.4) |
| Separated/divorced | 107 (11.2) |
| Single | 449 (52.1) |
| Widow/widower | 12 (1.3) |

**Table S2**

*Measures in Detail*

| Section | Descriptions |
| --- | --- |
| EFI-30 | The EFI-30 assesses the degree to which a person forgives a specific situation of offense (Enright et al., 2022). It consists of 30 items measured on a 6-point scale (from 1 [*strongly disagree*] to 6 [*strongly agree*])*.* The instrument includes three parts. (1) *Presentation:* The presentation includes four exploratory questions about an event (e.g., “How long ago was the offense?”)*.* (2) *Forgiveness Scale:* Its items are divided into six dimensions (five items per dimension): positive affect, negative affect, positive behaviour, negative behaviour, positive cognition, and negative cognition. The total scores of the scales are interpreted as the degree of presence of positive/negative feelings, cognitions, and behaviours towards the person who was unfair to the participant. In addition, the EFI-30 includes five pseudo-forgiveness questions; cases with a sum score ≥ 20 were removed. (3) *Forgiveness Item*: The one-item question (“To what extent have you forgiven the person?”) was measured on a 5-point Likert scale (from 1 [*not at all*] to 6 [*complete forgiveness*]). |
| Social desirability | The 18-item version of the Crowne and Marlowe Social Desirability Scale (MS-SDS; Gutiérrez et al., 2016) was used to assess social desirability. All items were measured on a dichotomous scale, either 0 (*false*) or 1 (*true*). To calculate the total score, 10 of the items were reverse-coded; the sum score was used in this study. The internal consistency of this scale was acceptable in this sample, Cronbach’s α = .69. |
| Demographics | Demographic variables included gender, sex, age, educational level, and country of origin (see Supplementary Materials, Table S1, for details). |

**Table S3**

*Correlations Between Subscales and the One-Item Forgiveness and Social Desirability Measures*

| EFI subscale | Pearson’s *r* | |
| --- | --- | --- |
|  | One-item forgiveness | Social desirability |
| Positive Affect | .34** | .08* |
| Negative Affect | -.29** | -.05 |
| Positive Behaviour | .34** | .06 |
| Negative Behaviour | -.31** | -.05 |
| Positive Cognition | .38** | .09** |
| Negative Cognition | -.32** | -.02 |

*Note*. **p* < .05; ***p* < .01.
